# A review of current progress in triple-negative breast cancer therapy

**DOI:** 10.1515/med-2020-0138

**Published:** 2020-11-14

**Authors:** Meiying Shen, Huawen Pan, Yuxia Chen, Yu Hang Xu, Weixiong Yang, Zhaojun Wu

**Affiliations:** Department of Mammary Gland, Maoming People’s Hospital, Maoming, 525000, China; Department of Spinal, Maoming People’s Hospital, Maoming, 525000, China; Department of Ultrasound, Maoming People’s Hospital, Maoming, 525000, China

**Keywords:** triple-negative breast cancer, PARP inhibition, immunotherapy, adjuvant therapy

## Abstract

Triple-negative breast cancer (TNBC) is a particularly aggressive subtype known for its extremely high drug resistance, progression, poor prognosis, and lack of clear therapeutic targets. Researchers are aiming to advance TNBC treatment worldwide. In the past 2–3 years, more positive results have emerged in the clinical research on TNBC treatment. Based on the results, several impressive drugs have been approved to benefit patients with TNBC, including the PARP inhibitors olaparib and talazoparib for germline BRCA mutation-associated breast cancer (gBRCAm-BC) and immunotherapy using the checkpoint inhibitor atezolizumab in combination with nab-paclitaxel for programmed cell death-ligand 1-positive (PD-L1+) advanced TNBC. Although neoadjuvant therapy has focused on combinations of systemic agents to optimize pathologically complete response, metastatic TNBC still has a poor prognosis. Innovative multidrug combination systemic therapies based on neoadjuvants and adjuvants have led to significant improvements in outcomes, particularly over the past decade.

## 1 Introduction

Triple-negative breast cancer (TNBC) is characterized by the absence of expression of the estrogen receptor, progesterone receptor, and human epidermal growth factor 2 (HER2) receptor [1]. Although the breast cancer (BC) molecular type is superficially defined by immunohistochemistry, the molecular subtypes and mechanisms are actually very complicated [2]. TNBC, accounting for approximately 15% of all BCs, is not sensitive to endocrine therapy and molecular-targeted therapy [3]; thus, surgery and systemic chemotherapy are the main treatment methods. The adjuvant therapy treatment regimen always includes anthracycline and/or paclitaxel in (Neo-) early TNBC; however, this treatment is often ineffective after recurrence or metastasis. Thereby, continuously searching for new targeted therapies and immunotherapies has gradually become a focus in the TNBC treatment in addition to developing new uses for old medicines. In recent years, great progress has been made in the treatment of TNBC. Notably, at the 2019 SABCS conference, a Chinese team presented the data on the treatment progress for TNBC that year. This article presents details of the latest progress in therapies for TNBC.

## 2 Targeted therapy: PARP inhibitors

In recent years, targeted treatment of TNBC has made some progress. BCs associated with germline mutations in BRCA1/2 account for 3–5% of cases. BRCA1/2-associated BC cases have biological features causing genomic instability and potential sensitivity to DNA damaging agents, including poly(ADP-ribose) polymerase (PARP) and platinum agents [4]. A meta-analysis has summarized 34 studies associated with BRCA genes, including 2,97,402 BC patients, and has shown that the prognosis of germline BRCA1/2 mutant BC is poor. Beyond the *BRCA1/2* genes, which are responsible for DNA damage repair, another important DNA damage repair pathway involves PARP-mediated repair of DNA single-strand breaks, which can lead to double-strand breaks that cannot be repaired in the process of DNA replication [5]. PARP inhibition can induce further DNA damage. If there is a *BRCA1/2* gene mutation and the DNA double-strand damage repair function is lost, inhibition of PARP enzyme function with a PARP inhibitor further hinders the possibility of DNA repair in tumor cells, thus accelerating the death of tumor cells, having a synthetic lethal effect, and implementing precise targeting [6,7]. PARP inhibitors provide further possible therapy options for BRCA mutant BC. To date, three main clinical trials on PARP inhibitors for TNBC have been performed, one of which includes promising results from the OlympiAD study presented at SABCS this year.

OlympiAD was a randomized, open-label, phase III trial evaluating olaparib monotherapy (300 mg tablets twice daily) compared with conventional chemotherapy. A total of 302 patients who had received two or fewer prior therapies in an advanced setting were randomized in a 2:1 ratio to olaparib or chemotherapy. After a median follow-up of 14.5 months, progression-free survival (PFS) was the primary endpoint. The results, first presented at ASCO in 2017, indicated significantly prolonged PFS with olaparib versus standard therapy (7.0 vs 4.2 months; hazard ratio (HR), 0.58; 95% CI, 0.43–0.8; *P* < 0.001); in addition, higher response rates were seen in the olaparib group (59.9% vs 28.8%). Olaparib was the first PARP inhibitor to demonstrate superior efficacy and tolerability than standard chemotherapy in *gBRCA*-mutated advanced BC. According to the results presented earlier, the FDA approved olaparib as the first PARP inhibitor for the treatment of this patient subgroup. Nevertheless, in the interim analysis, no difference in the overall survival (OS) was observed between the two groups [8]. OS values with the prolonged follow-up in the OlympiAD trial were reported at the 2019 SABCS conference. Among the patients who did not receive chemotherapy in an advanced stage, the results for the olaparib group were more significant than those for the chemotherapy group in terms of OS benefits. Unexpectedly, the median OS was 22.6 months in the chemotherapy group and 14.7 months in the control group. More remarkably, the 3-year OS was 40.8% versus 12.8% in the two groups, and no new safety events were observed in the extended follow-up [9]. Accordingly, a substantial proportion of patients with TNBC receiving olaparib therapy can attain long-term survival. Currently, research on PARP inhibitors for adjuvant therapy and neoadjuvant therapy, as well as for the prevention of BC is ongoing, including OlympiA (a phase III study) and GeparSixto study, and in the future, the results of these studies will show evaluating adjuvant therapy with olaparib for HER-2-/gBRCAm BC and exploring the value of a PARP inhibitor in neoadjuvant therapy, respectively.

EMBRACA is an open-label phase III trial that randomly assigned 431 patients in a 2:1 ratio to talazoparib at 1 mg daily or a standard single-agent therapy of the physician’s choice (capecitabine, eribulin, vinorelbine, or gemcitabine); 287 patients received talazoparib, and 144 patients received the standard treatment. All patients had gBRCA mutant, HER-2-negative advanced BC and received no more than three prior lines of chemotherapy in an advanced setting. Median PFS was the primary endpoint. The secondary efficacy endpoints included OS, objective response rate (ORR), clinical benefit rate at 24 weeks (the rate of complete, partial, or stable response), and the duration of the response. The median PFS was 3 months longer for talazoparib than for chemotherapy (8.6 vs 5.6 months; HR for disease progression or death, 0.54; 95% CI 0.41–0.71; *P* < 0.001), and the ORR was superior (62.6% vs 27.2%; HR, 4.99; *P* < 0.0001). An interim analysis of overall survival suggested a positive trend favoring talazoparib although these data are preliminary. Talazoparib, another PARP inhibitor, is a well-tolerated drug. The common adverse events (>10%) include anemia, fatigue, neutropenia, nausea, headache, and thrombocytopenia. Grade 3–4 hematologic adverse events occurred in 55% of patients versus 38% in the chemotherapy group, grade 3 or 4 anemia occurred in 39.2 vs 4.8%, and thrombocytopenia occurred in 14.7 vs 1.6%; however, grade 3 or 4 neutropenia was less frequent with talazoparib (20.9 vs 34.9%). PARP inhibitor drugs are a relatively nontoxic oral therapeutic option with many other advantages over conventional cytotoxic chemotherapy. The FDA has approved talazoparib for the treatment of metastatic BC patients carrying germline BRCA1/2 mutations, and a meta-analysis has confirmed that PARP inhibitors significantly delay the deterioration in the quality of life (HR, 0.40; 95% CI 0.29–0.54) [10].

A phase III study, BROCADE, randomized patients with gBRCAm-BC to carboplatin/paclitaxel with or without veliparib as a second-line treatment separately, and the results indicated higher PFS (14.5 vs 12.6 months, HR 0.71; *p* = 0.002); however, the OS did not differ significantly between groups (33.5 vs 28.2 months). Of note, the most common grade 3 toxicities were anemia (27 vs 17%), neutropenia (52 vs 50%), and thrombocytopenia (25 vs 15%) [11].

## 3 Immunotherapy

The immune system includes many immune checkpoints in the inhibitory signaling pathway, which are involved in regulating the persistence and the intensity of the immune response, maintaining autoimmune tolerance, and avoiding tissue damage [12,13]. Inhibition of immune checkpoints can reverse the microenvironment immunosuppressive state and enhance the function of tumor cell clearance [14,15]. In recent years, one major immune checkpoint therapy has involved a monoclonal antibody against programmed cell death-1 (PD-1)/programmed cell death-ligand 1 (PD-L1), which is expressed in a variety of tumor cells and immune cells. In tumor cells, PD-L1 binds its receptor PD1 on the surfaces of tumor-infiltrating lymphocytes and subsequently transmits immunosuppressive signals to TILs; inhibits T cell migration, proliferation, and secretion of cytotoxic mediators; and induces T cell depletion, thus limiting its killing effect on tumor cells. Ultimately, tumor cells successfully escape, whereas blocking the binding of PD1/PD-L1 may reverse this immune escape, enhance tumor immunity, and inhibit tumor progression [16]. In addition, using PD-1/PD-L1 immunotherapy has been demonstrated to be an effective treatment in clinical trials of patients with many tumors, and TNBC treatment has made some progress. Furthermore, tumors with a high mutational burden have superior responses to checkpoint inhibition, thus potentially explaining why TNBC treatment is the most advanced field, given the higher mutational burden in TNBC than in other BC subtypes [17]. We describe some trials in this article.

Pembrolizumab, a PD-1 inhibitor, has been used as a monotherapy to treat PD-L1-positive metastatic TNBC in the phase Ib KEYNOTE-012 trial. The results have revealed an overall response rate of 19%, with one complete response, four partial responses, and 26% patients with stable disease [18]. An ORR of 18.5% was achieved in metastatic TNBC patients, with a median OS of 11.2 months. Of total, 15.6% of the subjects enrolled in this trial were treatment naive.

In the phase II trial KEYNOTE-086, patients with metastatic TNBC were categorized according to PD-L1 expression and their history of metastatic treatment [19]. Cohort A included 170 patients with previously treated TNBC, regardless of PD-L1 expression, and the efficacy and safety of pembrolizumab were evaluated. An ORR of 4.7% was observed, a value lower than expected, and the PFS was similar in both the PD-L1-positive and negative cohorts (2.7 vs 1.9 months, respectively). Moreover, no significant difference was found in OS (8.3 vs 10 months in the PD-L1-positive and negative cohorts, respectively). Cohort B, including 84 patients who were PD-L1 positive on tumor cells or in the stroma and who had no prior metastatic treatment, experienced a higher ORR of 23.1%, and the median DOR was 8.4 months. The median PFS was 2.1 months, and the median OS was 16.1 months.

A total of 622 patients with advanced TNBC were randomized in a 1:1 ratio to pembrolizumab versus single-agent physician’s choice chemotherapy (capecitabine, eribulin, gemcitabine, or vinorelbine) as a second- or third-line therapy in the phase III KEYNOTE-119 trial. The official results have not been published, but a press release at the May 2019 ESMO announced that pembrolizumab did not meet its primary endpoint of OS [20]. KEYNOTE-522 study is the first prospective, randomized trial that enrolled 1174 patients receiving either neoadjuvant therapy or postoperative adjuvant therapy, assigned to pembrolizumab + chemotherapy group and placebo + chemotherapy group for TNBC in a 2:1 ratio. Pathological complete response and event-free survival were endpoints, and pCR values were notably 64.8 vs 51.2% between two groups in the first interim analysis and in the second interim analysis, and the results showed that the EFS rates were 91.3 and 85.3%, respectively. The results showed that platinum- containing neoadjuvant chemotherapy combined with pembrolizumab could significantly improve the pCR rate compared with chemotherapy 20.

Another immunotherapy study presented at the 2018 ESMO included IMpassion 130, a phase III registration study that randomly recruited 902 patients with metastatic or inoperable locally advanced TNBC, and evaluated nab-paclitaxel plus the PD-L1 inhibitor atezolizumab versus nab-paclitaxel plus placebo in patients as the first-line therapy [22]. Patients were stratified according to PD-L1, which was defined as positive with 91% staining of immune cells. The co-primary endpoints were PFS and OS in the ITT and PD-L1-positive population. The median follow-up was 12.9 months. The PFS was improved by just over 1.5 months in the ITT population with a combination of atezolizumab and nab-paclitaxel (7.2 months vs 5.5 months; HR, 0.80; 95% CI, 0.69–0.92; *P* = 0.002). However, PD-L1-positive patients showed a 2.5-month improvement with atezolizumab in PFS (7.5 months vs 5.0 months; HR, 0.62; 95% CI, 0.49–0.78; *P* < 0.001). OS in the ITT population was improved by approximately 4 months with atezolizumab, and the median OS was 21.3 versus 17.6 months in the atezolizumab group and placebo group, respectively (HR, 0.84; 95% CI, 0.69–1.02; *P* = 0.08). However, the difference in OS was much greater (10.5 months) in the PD-L1-positive population, and 54% of patients were alive at 2 years. Notably, the median OS was 25.0 versus 15.5 months (HR, 0.62; 95% CI, 0.45–0.86). The results of the second interim analysis of IMpassion130, presented at 2019 ESMO, were consistent with those of the first analysis [23]. Nab-paclitaxel plus the PD-L1 inhibitor atezolizumab was further confirmed to achieve clinically significant OS benefits in patients with primary PD-L1-positive metastatic TNBC. Based on the IMpassion130 results, the FDA granted accelerated approval of atezolizumab in combination with nab-paclitaxel for the treatment of patients with unresectable locally advanced or metastatic PD-L1-positive TNBC. Nonetheless, we are also looking forward to the outcomes of IMpassion131, a similar phase III trial designed to study atezolizumab + paclitaxel versus paclitaxel + placebo as a first-line therapy in TNBC to determine the difference of atezolizumab used in first-line versus second-/third-line of treatment.

## 4 Chemotherapy: new uses of old drugs

Chemotherapy is an accepted treatment for early-stage TNBC, but no specific regimens are used for improving prognosis [24,25]. In recent years, studies have increasingly sought better ways to treat this BC type. Neoadjuvant chemotherapy is the standard of care for locally advanced or inoperable TNBC. Patients with TNBC, as opposed to those with the luminal subtypes, are more likely to achieve a pCR with neoadjuvant chemotherapy [26]. A major advantage of this approach is its ability to predict survival according to the presence or the absence of a pCR at the time of surgery and to tailor adjuvant therapy. However, it can be used to screen patients who are prone to metastasis and recurrence, so that further intensive treatment can be provided for those who do not meet this endpoint. This process is critical, because these patients have a relapse risk 6 to 9 times higher than those of patients achieving pCR [27]. Neoadjuvant chemotherapy should be optimized, reasonable, sufficient, and standard (six to eight phases) to enable higher pCR and decrease treatment or ladder treatment in postoperative adjuvant therapy. For patients who do not reach pCR, intensive treatment is required, such as capecitabine treatment, which can be used as a second-line intensive treatment regimen.

Capecitabine is an oral prodrug that is enzymatically converted to 5-flfluorouracil in the body. The FDA initially approved this agent (marketed as Xeloda) in 1998 for use in patients with metastatic HER-2-negative BC that had progressed after administration of both anthracycline and taxane [28,29,30,31,32,33]. In recent years, several randomized clinical trials have estimated the clinical value of adding capecitabine as adjuvant therapy for early BC (including TNBC) although the studies have generated conflicting conclusions [34,35,36,37]. Because TNBC has the highest metastasis and recurrence rates and the lowest survival rate among BC subtypes [38], investigating the clinical value of adjuvant addition of capecitabine in early-TNBC patients and determining the targeted subgroup that can benefit most are crucial.

The FinXX exploratory randomized clinical trial examined 1,500 women in Finland and Sweden between January 27, 2004, and May 29, 2007; 747 women received three cycles of docetaxel followed by three cycles of cyclophosphamide, epirubicin, and fluorouracil, and 753 women received three cycles of docetaxel plus capecitabine followed by three cycles of cyclophosphamide, epirubicin, and capecitabine according to a random allocation [39]. The primary endpoint was recurrence-free survival (RFS), and the median follow-up time was 10.3 years. No significant difference was observed in RFS or OS between the groups (HR, 0.88; 95% CI, 0.71–1.08; *P* = 0.23; and HR, 0.84, 95% CI, 0.66–1.07; *P* = 0.15), yet BC-specific survival tended to favor the capecitabine group (HR, 0.79; 95% CI, 0.60–1.04; *P* = 0.10), and a comparison of the subgroups defined by cancer steroid hormone receptor status indicated that the capecitabine group was superior in terms of RFS and OS in the subset of patients with TNBC (HR, 0.53; 95% CI, 0.31–0.92; *P* = 0.02; and HR, 0.55, 95% CI, 0.31–0.96; *P* = 0.03). This trial has concluded that patients with TNBC have favorable survival outcomes when treated with the capecitabine containing regimen, but the results must be cautiously interpreted because of the exploratory subgroup analysis design. Nonetheless, concluding that capecitabine has no role in the adjuvant treatment of early BC may be premature, and the ongoing trials may provide more guidance.

In the other open label trial, CREATE-X, reported by the Japanese Breast Cancer Research Group (JBCRG) in 2015, also called JBCRG-04, all 910 eligible patients had HER-2-negative stage I to IIIB BC and had residual disease in the breast or lymph nodes after neoadjuvant chemotherapy. The participants were randomized to either capecitabine (*n* = 455) or no additional chemotherapy (*n* = 455), to identify whether patients who had residual tumors after neoadjuvant chemotherapy with both anthracycline and a taxane, and received sequential adjuvant administration of capecitabine, which would show a survival benefit. Disease-free survival (DFS) was the primary endpoint, and OS was a secondary endpoint; both were improved in the TNBC cohort with six to eight cycles of the adjuvant capecitabine. After 5 years of follow-up, the DFS was 82.8% in the capecitabine arm versus 74% in the control arm (HR, 0.7; 95% CI, 0.53–0.93; *P* = 0.005). Moreover, the OS was 89.2% in the capecitabine arm, but 83.9% in the control arm (HR, 0.60; 95% CI, 0.40–0.92; *P* = 0.001). Similarly, the subset of patients with TNBC demonstrated a statistically significant improvement in DFS (HR, 0.58; 95% CI, 0.39–0.87). Hand-foot syndrome, the most common adverse reaction to capecitabine, occurred in 73.4% of the patients in the capecitabine group [40]. The results of the CREATE-X trial had prompted most clinicians to treat early-stage TNBC with neoadjuvant chemotherapy to determine who should receive capecitabine.

The GEICAM/2003–11_CIBOMA/2004–01 trial is an open-label, randomized phase III study that explored extended adjuvant capecitabine after completion of standard chemotherapy in patients with early TNBC [41]. Early-TNBC patients who had prior anthracycline- and/or taxane-containing chemotherapy were randomly allocated to either capecitabine (*n* = 448) or observation (*n* = 428). The median length of follow-up was 7.3 years, and the primary endpoint was DFS between groups. The DFS was not significantly prolonged in the capecitabine group versus the observation group (HR, 0.82; 95% CI, 0.63–1.06; *P* = 0.136). Disappointingly, the GEICAM-CIBOMA study did not show a statistically significant improvement in DFS with the addition of capecitabine to standard (neo)adjuvant chemotherapy for operable TNBC, as reported at the 2019 SABCS.

The China Breast Cancer Clinical Study Group (CBCSG) combined efforts of 35 study centers and addressed all kinds of difficulties to contribute to the CBCSG010 trial, the largest phase III randomized clinical trial performed in China to date, assigned patients separately to the observation group (three cycles of paclitaxel combined with capecitabine and three sequential cycles of capecitabine combined with epirubicin + cyclophosphamide) and control treatment (three cycles of paclitaxel and three sequential cycles of cyclophosphamide, epirubicin, and fluorouracil) in the adjuvant treatment phase, and compared the safety and efficacy of two schemes. The median follow-up was approximately 5 years; the DFS was 86.3% in the treatment group versus 80.2% in the control group in TNBC (HR = 0.66); the risk of recurrence was reduced 34%; and the RFS was 89.5 versus 82.9% in the treatment group and the control group, respectively (HR = 0.58). Subgroup analysis indicated that patients with T2/T3 tumor stage, positive lymph nodes, level III histological grade, and high levels of Ki-67 show apparent benefits in DFS and OS. On the basis of these results, capecitabine can be combined with a regimen containing anthracycline and paclitaxel in the early adjuvant therapy stage to improve prognosis [42]. Future CBCSG010 research will further explore populations that would benefit by combining the Fudan four classification standard of TNBC [43]. Because all drugs in this research have been approved and are covered by the national medical insurance in China, more patients with TNBC could get longer and better survival benefits. Ultimately, toxicity should be noted.

## 5 Conclusions: challenges and forecast

Surprising results in 2019 indicated that PD-1 and PD-L1 inhibitor treatment may open doors for further immunotherapy treatment. In approximately 40% of patients who are PD-L1 positive, atezolizumab + nab-paclitaxel can be used as a first-line therapeutic regimen to increase survival benefits. We expect to see more research in this domain, such as using atezolizumab in neo-/adjuvant therapy to improve the prognosis of TNBC and searching for biomarkers. Accordingly, PARP inhibitors are playing an increasing role for patients with gBRCAm, and clinical phase III data demonstrate that these patients could experience the benefit of limiting disease progression Moreover, the outcomes in terms of adverse events and quality of life are superior to those with chemotherapy. Undeniably, the strategy to escalate chemotherapy with capecitabine can prolong a certain survival rates and lower recurrence rates in some early TNBC patients, thus benefiting those patients who cannot afford expensive and currently still unattainable medicine.

Notably, determining the TNBC phase is very important for clinical diagnosis and treatment. If a patient remains in an early stage, then only chemotherapy can achieve a high-quality outcome without additional toxicity. However, additional steps must be taken, such as identifying the status of BRCA and PD-L1 in locally advanced or metastatic TNBC, to determine whether a PARP inhibitor or atezolizumab might be needed.
